# Scion and Rootstock Differently Influence Growth, Yield and Quality Characteristics of Cherry Tomato

**DOI:** 10.3390/plants9121725

**Published:** 2020-12-07

**Authors:** Rosario Paolo Mauro, Michele Agnello, Andrea Onofri, Cherubino Leonardi, Francesco Giuffrida

**Affiliations:** 1 Dipartimento di Agricoltura, Alimentazione e Ambiente, University of Catania, Via Valdisavoia, 5-95123 Catania, Italy; micheleagnello@hotmail.it (M.A.); cherubino.leonardi@unict.it (C.L.); francesco.giuffrida@unict.it (F.G.); 2 Dipartimento di Scienze Agrarie, Alimentari e Ambientali, University of Perugia, Borgo XX Giugno, 74-06121 Perugia, Italy; andrea.onofri@unipg.it

**Keywords:** *Solanum lycopersicum* L., grafting, yield, fruit quality, environmental variance, rootstock-scion combination

## Abstract

Grafting is a valuable tool for managing problems of tomato soil-borne pathogens and pests, but often generates unpredictable effects on crop yield and product quality. To observe these rootstocks-induced changes, experimental designs including many rootstock-scion combinations are required. To this end, a greenhouse experiment was conducted on 63 graft combinations, involving seven cherry tomato scions grouped in large, medium and small-fruited, and eight rootstocks with different genetic backgrounds (crosses between *Solanum lycopersicum* and *S. habrochaites* or *S. peruvianum* or *S. pimpinellifolium*, plus an intraspecific hybrid), using ungrafted controls. The response of the graft partners was firstly analyzed individually using the environmental variance (σ^2^_E_), then by grouping them by classes. When analyzed individually, the scion genotype influenced fruit L*, b*, shape index, total soluble solids (TSS) and its ratio with tritatable acidity (TSS/TA), whereas plant growth and yield were unpredictable. After clustering the graft partners, some of these responses were attributable to the imposed classes. The *S. habrochaites*-derived hybrids maximized plant biomass, unlike the *S. pimpinellifolium* ones. Both classes reduced fruit biomass in small- and medium-fruited scions (by 11 and 14%, respectively). The *S. habrochaites* and *S. peruvianum* hybrids reduced a* and TSS, whereas promoted TA. L-ascorbic acid was reduced by grafting (from −23 to −45%), in the *S. pimpinellifolium* group too, indicating, even in low vigor rootstocks, a dilution effect worsening this nutraceutical trait of tomatoes.

## 1. Introduction

Vegetable grafting was initially promoted to meet the restrictions to soil disinfestation against soil-borne pathogens and pests, but over time, it has become a strategic tool for improving the crops’ performances under a wide array of suboptimal growth conditions [1,2,3], becoming a cornerstone of the modern sustainable horticulture. Nowadays, grafting is recognized as a pivotal means to modify the vegetative vigor and maximize the yield of several horticultural crops [4], but such increases can be accompanied by variable effects on fruit quality [5].

Tomato (*Solanum lycopersicum* L.) is one of most important greenhouse crops in the Mediterranean Basin [6,7], for which grafting is considered a standard practice in commercial greenhouse cultivations [8]. An improved yield is commonly reported in tomato grafted onto appropriate rootstocks as a result of an increased fruit size or number of fruits per plant, the former feature typically recognized also in scions with small-sized fruits, such as cherry tomato [5,9]. It has been reported that vigorous rootstocks tend to increase fruit yield probably by enhancing water and nutrient uptake and transport to the scion [10]. As a consequence of the central contribution of both processes to fruit metabolism, several authors have noticed rootstock-mediated effects on many tomato traits such as shape [11], color [12], texture [13], taste and aroma components [14,15] or ascorbic acid content [16]. However, these effects have been even conflicting among authors, making impossible to draw sound conclusions about the overall effects of this technique on tomato quality. This is primarily due to the existing complex interactions involving the genetic background of the grafting partners and the surrounding growth environment in determining the tomato phenotype [17]. Moreover, the researches in this field have been conducted using different scion cultivars and fruit typologies, making impossible to compare indiscriminately the relative findings.

To date, the most popular approach has been to examine the effects of different rootstocks on one or few scions. Although giving useful information, this may not consider the typical characteristics of the scion, which actively exchanges photosynthates and metabolic messengers with the rootstock [17]. To observe the rootstocks-induced changes in tomato yield and fruit quality without missing the active role of the scion and rootstock characteristics, large experimental designs including many rootstocks-scion combinations are required. With this in mind, the present experiment was designed to examine, in a large set of grafting combinations (7 scions × 8 rootstocks, plus ungrafted controls), the effects of grafting on growth, yield and quality of greenhouse cherry tomato, with particular focus at elucidating (i) the possible predominance either of the scion or of the rootstock in influencing the response of the grafted plant, and (ii) whether these results could be generalized on the basis of the fruit size of the scion or of the rootstock genetic background.

## 2. Materials and Methods

### 2.1. Experimental Site and Management Practices

The experiment was conducted in Sicily, Southern Italy (36°59′11.9″ N; 14°21′35.8″ E; 40 m a.s.l.), during the 2017–2018 growing season, in a greenhouse of 2400 m^2^ with a sandy soil and an electrical conductivity of the irrigation water around 1500 µS cm^−1^. The climate of the area is semi-arid Mediterranean, with mild winters and hot, dry summers. The greenhouse hosting the crop had a steel tubular structure and windows along the sides, covered before transplanting with a 200 µm thick ethylene vinyl acetate (Agriplast s.r.l., Modica, Italy). The soil along the plant rows was covered with a black polyethylene film (40 µm tick) few days before transplanting. Tomato seedlings were transplanted on 5 October 2017 at the stage of three true leaves, adopting a 0.40 × 1.00 m rectangular format (2.5 plants m^−2^), and trained at two stems per plant up to 17 May 2018 (224 days after planting, overall 11 fruit harvests), with a production of 11 trusses per stem, as is usual for the reference area. Plants were obtained from a specialized nursery, where the splice-grating technique was applied, followed by the application of a plastic clip and stick to secure the graft union. Before the start of the experiment, plants were selected for homogeneous size and apparent health characteristics. Once transplanted, all plants were managed according to the same standard commercial practices, receiving 25.2 g N, 18.7 g P_2_O_5_, 22.8 g K_2_O, 28.4 g MgO and 0.5 g Fe per plant. Drip irrigation was effected when the accumulated evapotranspiration outside the greenhouse reached 40 mm (estimated with the Penman–Monteith equation). All meteorological data were obtained daily from SIAS (Servizio Informativo Agrometeorologico Siciliano). Pests control was performed as per local custom.

### 2.2. Plant Material and Experimental Design

The genotypes used in the experiment are reported in Table 1. The scion cultivars were chosen on the basis of their different fruit size, among those most used in the reference area. From previous information, productive data of ungrafted plants under comparable growth conditions for several cherry tomato cultivars were acquired (Giuffrida, unpublished data). The seven selected scions were then divided into three classes, namely large-fruited (average fruit diameter 34–36 mm), medium-fruited (31–33 mm) and small-fruited (28–30 mm). Eight genotypes representing the majority of the rootstocks available for tomato were used, of which four were *S. lycopersicum* × *S. habrochaites* hybrids, two *S. lycopersicum* × *S. peruvianum*, one *S. lycopersicum* × *S. pimpinellifolium* and one intraspecific tomato hybrid. Ungrafted plants of each scion were used as controls. A randomized complete blocks design was used with 63 grafting combinations and three replicates for each combination. Each experimental unit contained nine plants (net of borders).

### 2.3. Plant Growth and Yield Variables

The dry biomass of the different plant fractions (stems, leaves and fruits) was obtained by drying samples in a thermoventilated oven (Binder, Milan, Italy) at 70 °C, until constant weight was reached. The fruit harvests were effected by hand, when the last two fruits of each truss were at the red stage (stage F), according to Gautier et al. [18]. The dry weight of leaves was determined at each leaf removal and stem dry weight was determined soon after the last harvest, whereas at each harvest, the number of fruits per truss and the fresh and dry weight of the fruits was determined. From the original dataset, whole plant, vegetative fraction and fruit dry biomass, as well as the harvest index (HI) were calculated, this last variable as the ratio among fruits and total aboveground biomass of the plant.

### 2.4. Fruit Quality Traits

Within 2 h of harvest, the fruits were transported to the laboratory for further processing and determination of the main quality traits [9]. To this end, the fruits were deprived from rachis then weighed to determine their mean weight, whereas the fruit shape index was calculated as the ratio between longitudinal and transversal diameter, both determined through a digital caliper. The chromatic coordinates were measured as described by McGuire [19] on four points per fruit along the equatorial axis, through a tristimulus Minolta Chroma Meter (model CR-200, Konica Minolta, Inc., Tokyo, Japan), calibrated with a standard white tile (UE certified) with illuminant D65/10°, measuring color in terms of lightness (L*), green-red axis (a*) and blue-yellow axis (b*). Fruit firmness was measured through a texture analyzer (Stable Micro Systems model TA-XT2), expressing the force needed to give a 2 mm deformation of the fruits along their polar axis among two steel plates. Total soluble solids (TSS) content was determined through a digital refractometer DBX-55° (Atago Co., Ltd., Tokyo, Japan) provided with an automatic temperature compensation system. Tritatable acidity (TA) was determined by neutralization of the free acids with a titration solution of 0.1 N NaOH up to the changing color of phenolphthalein. The results were then expressed as mg L^−1^ of citric acid equivalents. For the L-ascorbic acid (AsA) determination, 0.1 g of freeze-dried material was extracted in 2 mL of H_3_PO_4_ 0.05 N through sonication for 6 min. After centrifugation at 4 °C for 15 min at 13,000 g, the supernatant was collected, filtered through 0.45 µm nylon filters and analyzed by HPLC-UV (λ = 245 nm) equipped with an autosampler. The mobile phase was KH_2_PO_4_ buffer at 2.3 pH, and a reverse phase C18 column was used, with a 0.5 mL min^−1^ flow rate. Peak areas were converted to ascorbic acid through a standard curve prepared with L-ascorbic acid (Sigma-Aldrich, St. Louis, MO, USA). All quality determinations were effected on the first, sixth and eleventh trusses, i.e., on trusses harvested on 18 December, 28 March and 16 May, to cover the whole period of meteorological conditions during the growth cycle.

### 2.5. Statistical Procedures

All collected and calculated data were firstly subjected to Shapiro–Wilk and Levene’s tests, in order to check for normal distribution and homoscedasticity, respectively, then to a factorial ‘scion × rootstock’ (‘S × R’) analysis of variance (ANOVA), according to the experimental layout adopted in the greenhouse. Percentage data were submitted to arcsin-square root transformation before the ANOVA (untransformed data are reported and discussed), whereas multiple mean comparisons were performed through Tukey’s honestly significant difference (HSD) test (*p* ≤ 0.05). After this first step, the means for all ‘scion × rootstock’ combinations were used to parameterize a mixed model, where the main effects were assumed as fixed and the interaction was assumed as random. Consequently, environmental variances (σ^2^_E_) were calculated for the scions and rootstocks under study in relation to each response variable, according to Piepho [20]. Environmental variances are often used to assess the stability of genotypes across environments and, in our setting, they were used to measure the variability of performances for each scion across rootstocks and, conversely, for each rootstock across scions. A low environmental variance implies that the performances of each rootstock/scion tend to be not influenced by the graft partner. To better elucidate the variability between and within scion classes [large-fruited (one genotype), medium-fruited (four genotypes) and small-fruited (two genotypes)] and rootstock classes [*S. habrochaites* (four genotypes), *S. peruvianum* (two genotypes), *S. pimpinellifolium* (one genotype) *S. lycopersicum* (one genotype) and ungrafted], a third model was fitted to the observed data, where the ‘scion class’ (‘S_class_’) and ‘rootstock class’ (‘R_class_’), together with their interaction (‘S_class_ × R_class_’) were included as fixed effects, while the nested effects of rootstock and scion within ‘S_class_’, ‘R_class_’ and ‘S_class_ × R_class_’ were added as random effects, in order to be able to assess the significance of the differences between classes, based on within-class variability. Additionally, in this case, Tukey’s (HSD) test (*p* ≤ 0.05) was performed for the separation of classes means.

## 3. Results

### 3.1. Plant Growth and Developmental Variables

**Figure 1:**
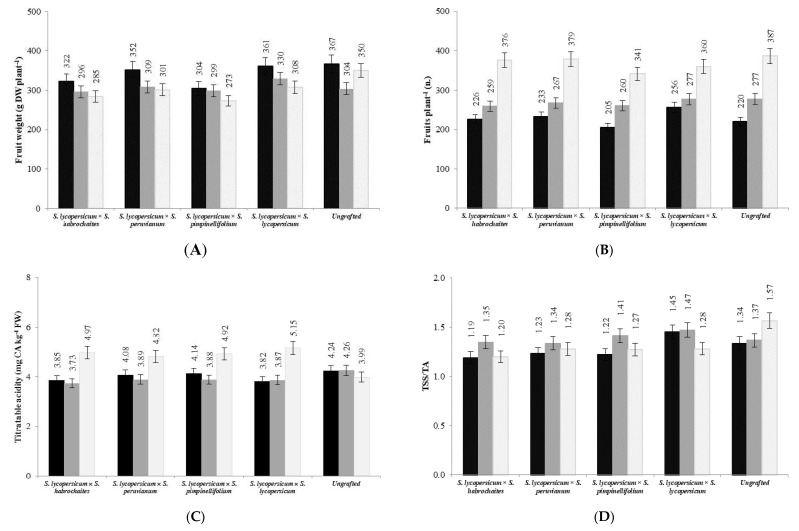
Fruit biomass (**A**), number of fruits per plant (**B**), tritatable acidity (**C**) and TSS/TA ratio (**D**) for the ‘scion class × rootstock class’ combinations. Black bars: large-fruited scion; dark grey bars: medium-fruited scions; light grey bars: small-fruited scions.

The plant growth performances were affected by scion (‘S’), rootstock (‘R’) along with their interaction (‘S × R’) (Table 2). For plant and vegetative biomass, ‘S’ showed the highest incidence on total variance, whereas for fruit biomass and harvest index (HI), the ‘S × R’ interaction proved to be the main source of variation. Among the scion cultivars, ‘5525′ highlighted the lowest environmental variance (σ^2^_E_) values for both plant and vegetative biomass, with an opposite response recorded in ‘Eletta’ (Table 3). ‘Beka’ and ‘Caprice’ showed extreme σ^2^_E_ values with reference to fruit biomass, whereas for HI the highest variability was recorded in ‘Dreamer’ and ‘Caprice’. With reference to rootstocks, ‘Pittam’ showed the highest σ^2^_E_ for plant and vegetative biomass, whereas for the same variables the lowest values were recorded in ‘Dynafort’ and in the ungrafted control, respectively (Table 4). This last showed also the highest σ^2^_E_ in relation to fruit biomass, whereas the opposite was recorded in ‘Kaiser’. Differently, ‘Interpro’ and ‘Bental’ proved the highest σ^2^_E_ related to HI (Table 4). When rootstock classes were considered, the *S. pimpinellifolium* rootstock displayed the least plant, vegetative and fruit biomass among the grafted plants, whereas the *S. habrochaites* hybrids proved the least HI value (Table 5). The significant ‘S_class_ × R_class_’ interaction highlighted that, in all grafting combinations, fruit dry biomass progressively decreased passing from large- to medium- then to small-fruited scions (from 335 to 308 then to 292 g plant^−1^, on average) (Figure 1A). In contrast, in the ungrafted controls, fruit biomass significantly decreased passing from large- to medium-fruited scions (from 367 to 304 g plant^−1^, −8%) then increased in the small-fruited ones (350 g plant^−1^, +15%) (Figure 1A).

### 3.2. Yield and Related Components

Yield and its components were all significantly affected by the main factors and their interaction, with ‘S’ being by far the main source of experimental variation (Table 2). Concerning the scion cultivars, ‘Creativo’ and ‘Dreamer’ showed the least σ^2^_E_ values related to yield and number of fruits per plant, respectively, whereas ‘Porpora’ proved the highest σ^2^_E_ for both yield and mean fruit weight (Table 3). ‘Caprice’ showed the highest σ^2^_E_ for the number of fruits per plant, while also showing the lowest one for mean fruit weight. Among the rootstocks, the highest σ^2^_E_ was recorded in ‘Optifort’ (yield) and ‘Kaiser’ (fruits per plant and mean fruit weight), whereas ‘Mozart’, ‘Tawa’ and the ungrafted test gave the lowest σ^2^_E_ for mean fruit weight, fruits per plant and yield, respectively (Table 4). When clustered according to their main characteristics, the large-fruited scion outcompeted the other groups in terms of yield and mean fruit weight (144.3 t ha^−1^ and 30.4 g, respectively), whereas the opposite was recorded for the small-fruited scions, with the medium-fruited ones always showing an intermediate behavior (Table 5). Regarding the rootstock groups, all the interspecific hybrids enhanced the mean fruit weight when compared to the ungrafted controls (from 19.7 to 21.7 g, on average, +10%) (Table 5). The significant ‘S_class_ × R_class_’ interaction highlighted that, by decreasing the fruit size, there was a significant increase in the number of fruits per plant. For this variable the least variations were recorded passing from small- to medium-fruited scions grafted onto the intraspecific hybrids (from 256 to 277 fruits plant^−1^, +8%) and passing from medium- to large-fruited scions grafted both onto the *S. pimpinellifolium* and the intraspecific combination (from 260 to 341 and from 277 to 360 fruits plant^−1^, respectively) (Figure 1B).

### 3.3. Fruit Chromatic Coordinates and Carpometric Variables

All the chromatic coordinates and carpometric variables were significantly affected by ‘S’, whereas the ‘S × R’ interaction affected fruit redness (a*) (Table 2). ‘Porpora’ showed the highest σ^2^_E_ values in relation to L* and b* and the least ones for a*, whereas low σ^2^_E_ values were recorded for ‘5525′ (b*) and ‘Caprice’ (L* and fruit firmness), with ‘Dreamer’ and ‘Creativo’ also showing the highest variability for a* and fruit firmness, respectively (Table 3). When rootstock cultivars were concerned, ‘Kaiser’, ‘Bental’ and ‘Optifort’ maximized the variability of the chromatic coordinates L*, a* and b*, respectively, whereas the lowest σ^2^_E_ values were recorded in the ungrafted control (L*), ‘Optifort’ (a*) and ‘Kaiser’ (b*) (Table 4). Moreover, fruit firmness variability peaked in the ungrafted control, whereas the rootstocks ‘Bental’ and ‘Mozart’ proved the least variability related to fruit firmness (Table 4). Considering the groupings of the grafting partners, almost all the chromatic coordinates and carpometric variables were unaffected either by ‘S_class_’ or ‘S_class_ × R_class_’, whereas both the *S. habrochaites* and *S. peruvianum* hybrids generated a significantly lower a* coordinate of the fruits (12.5) when compared to the intraspecific hybrid class (14.0) (Table 6).

### 3.4. Compositional Variables

The fruit compositional variables were significantly affected by ‘S’ and, in the majority of cases, by ‘R’ too, with the former factor being always the main source of variation (Table 2). In contrast, fruit TA was not responsive to ‘R’, whereas the ‘R × S’ interaction significantly affected only TA and AsA content. Among the scion cultivars, ‘Porpora’ showed the lowest σ^2^_E_ for TA and the highest one for AsA, whereas ‘5525′ highlighted the lowest σ^2^_E_ for both TSS and AsA (Table 3). ‘Dreamer’ evidenced the highest variability for TSS and the lowest one for TSS/TA, whereas ‘Beka’ and ‘Caprice’ were characterized by the highest σ^2^_E_ values related to TA and TSS/TA, respectively. Regarding the rootstock cultivar, the highest variability related to the taste descriptors, namely TSS, TA and their ratio, were recorded in ‘Optifort’, ‘Mozart’ and in the ungrafted control, respectively, whereas the lowest ones in the ungrafted control (TSS) and ‘Bental’ (TA and TSS/TA) (Table 4). In contrast, the extreme σ^2^_E_ values related to AsA content were recorded in ‘Pittam’ (the lowest) and in the self-rooted control. When grafting classes were concerned, the small-fruited scions were characterized by the highest TSS (Table 6), whereas a significant ‘S_class_ × R_class_’ interaction emerged for the other taste variables (Figure 1). Indeed, considering the TA, the ungrafted group did not significantly differ in relation to the scion typology, whereas the same variable, when compared to the other scion groups, significantly increased in the small-fruited scions, especially when grafted onto the *S. habrochaites* and onto the intraspecific hybrids (by 31 and 34%, respectively) (Figure 1C). Contrarily, the TSS/TA significantly increased with the decreasing fruit size in the ungrafted scions (passing from 1.34 to 1.57), whereas in the *S. habrochaites*, *S. pimpinellifolium* and the intraspecific classes, TSS/TA dropped passing from medium- to small-fruited scions (from 1.41 to 1.29, on average) (Figure 1D). The AsA content of tomatoes did not differed among scion typologies, but peaked in the ungrafted controls (305.6 mg kg^−1^ FW) (Table 6). Considering the other graft classes, this variable significantly decreased in the intraspecific hybrids (236.8 mg kg^−1^ FW, −23%) and in the *S. peruvianum* and *S. pimpinellifolium* hybrids (191.5 mg kg^−1^ FW, on average), with the *S. habrochaites* class showing the least AsA content (167.8 mg kg^−1^ FW, −45%) (Table 6).

## 4. Discussion

Under the experimental conditions in which we operated, ‘S’ explained the highest variability of both whole plant and vegetative biomass, whereas the influence of both ‘R’ and ‘R × S’ prevailed on harvest index. When the grafting part classes were considered, the scion group did not influence the growth and developmental variables, which, instead, were under the influence of the rootstock genetic background. It has been suggested that the improved plant growth in response to grafting flows from a greater root development, enabling plants for a better absorption of water and minerals from the growth substrate, thus maximizing the photosynthetic gain of the scion [1,21]. Among the rootstock classes considered in the present experiment, the *S. habrochaites* hybrids maximized both whole plants and vegetative biomass accumulation (with a 10 and 22% increase, respectively, when compared to the ungrafted class), whereas a significant reduction of both variables was recorded in the *S. pimpinellifolium* class in comparison to the most vigorous rootstocks (by 11 and 13%, on average). Accordingly, among the studied rootstocks, ‘Dynafort’ (*S. lycopersicum* × *S. pimpinellifolium*) proved to have the lowest plant and vegetative biomass and related σ^2^_E_ values, meaning that its tendency to reduce tomato plant growth was poorly affected by the scion cultivar. *S. habrochaites* is a highly vigorous species, adapted to thrive in a wide latitudinal distribution, showing characteristics promoting growth in grafted tomato [22,23]. On the other hand, *S. pimpinellifolium* is the closest wide relative to the cultivated tomato, and has been used to improve tomato resistance to several biotic stressors [24] or fruit quality traits [25]. Recently, Mata-Nicolás et al. [26], in a wide *Solanum* germplasm collection including *S. pimpinellifolium* and *S. lycopersicum*, found that the *S. pimpinellifolium* accessions were the least vigorous in terms of stem width and leaf length. Therefore, our results suggest that this low vigor tendency could be at the base of the low growth induction in grafted tomato scions. Interestingly, these two highly differentiated rootstock classes in terms of plant growth performances shared a decreased fruit biomass production when compared to the other graft combinations (−10%, on average). Concerning the *S. habrochaites* class, the reduction of fruit biomass acted to significantly lower the harvest index. This suggests that the enhanced plant growth was accompanied by an increased sink strength of the vegetative fraction. Indeed, it has been reported that vigorous rootstocks may act as additional sinks, exacerbating the competition for photosynthates allocation with other plant fractions [27]. The ‘S_class_ × R_class_’ interaction revealed that the fruit size of the scion contributed to buffer the sink strength of the rootstock. Indeed, when the ungrafted control class was considered, both large- and small-fruited scions showed the highest fruit biomass, whereas in three groups (*S. habrochaites*, *S. peruvianum* and intraspecific hybrids) grafting penalized the fruit biomass of both medium- and small-fruited scions, with the large-fruited one always showing the highest fruit biomass value. In this respect, the large-fruited class of our experiment comprised only one cultivar (‘Porpora’); for this reason, we are aware that larger experimental classes would be needed to parameterize the possible relationship we noticed among fruit size and biomass in grafted tomato plants. Interestingly, this reduction was restrained by the *S. pimpinellifolium* class, which conferred the least vegetative vigor to the scions. Recently, Grieneisen et al. [28], in a meta-analysis involving a large set of literature data, reported that, in the majority of the cases examined, tomato grafting had no effect (58% of the cases) or a negative effect (6%) on fruit yield. For the authors, it was impossible to separate the effects of the scion cultivar to explain the yield response included in the dataset. This is in agreement with our results, since ‘S’ was by far the main source of yield variation, followed by the ‘S × R’ interaction, meaning that under unstressed growth conditions like those of our experiment, the centrality of the rootstock per se in determining yield is at least partially lost. This implies that despite its central importance, the yield outcome of tomato grafting still remains poorly predictable and to be accessed case by case. However, when clustered by class in the ANOVA, the yields we recorded were proportional to the fruit size of the scions, denoting that medium- and small-fruited cherry tomatoes put a penalization on crop yield when grafting is concerned.

According to Schwarz et al. [13] and Kyriacou et al. [5], the mean fruit weight is one of the primary traits that is influenced by grafting. In our study, beyond the largely prevalent effect of ‘S’, the average fruit weight was significantly affected by ‘R’ and ‘R × S’ too, whereas all the interspecific hybrid classes promoted such trait, without interactive effects.

When positive, higher a* values refer to an increased intensity of the red hue, representing in tomato the top contributor to the lycopene-derived color [29]. In red-ripe tomatoes, both a* and b* have positive values, but the higher the b* value the more perceived color turns to orange, through progressive yellow addition. Higher L* values represent a transition toward lighter colors, potentially representing a deterioration of tomato pigmentation too. Graft-induced worsening of tomato color has been sometimes reported as a result of a reduced carotenoid concentration by grafting onto vigorous rootstocks [12,30], though this finding has not always been confirmed [13]. In the present study, the fruit chromatic coordinates were mostly ‘S’-dependent, though a significant ‘R × S’ interaction was recorded for a*. Accordingly, for all these chromatic coordinates, the scion genotypes proved lower σ^2^_E_ values than the rootstock ones, with a* showing higher σ^2^_E_ values than L* and b*. ‘Porpora’ (large-fruited) and ‘Caprice’ (small-fruited) exhibited the highest stability related to a*, meaning that the ability of the scion to superimpose the red hue was independent from the fruit size. In contrast, ‘Bental’ and ‘Pittam’ (both deriving from *S. peruvianum*) showed the highest σ^2^_E_, and the ANOVA by classes revealed that they acted to reduce a*, together with *S. habrochaites* hybrids (−9%, on average). It is interesting to note that both rootstock classes were characterized by the highest vegetative biomass, so we cannot exclude that their depressive effect sometimes recorded on a* was not associated with a heavier fruit shading and subsequent lower fruit temperature, since both optimal light and temperature are key promoters of lycopene accumulation in tomatoes [31,32].

Fruit shape modifications accompanying the increased fruit size have been reported in grafted tomato [5]. However, we did not observe any shift in fruit shape in response to grafting, though some fruit weight increases were observed. This indicates a strong maintenance of the typical shape in all the studied scions, regardless of the grafting combinations, consistent with the known poor dependence of this trait on non-genetic factors [33].

Texture, often described through firmness, is a key quality trait of tomato, as it influences postharvest transportability, shelf-life and even flavor perception [34,35]. Although tomato firmness is often reduced by vigorous rootstocks, the results in this regard are sometimes contrasting [36,37]. In our experiment, fruit firmness was not affected by ‘R’ nor by ‘S × R’, being ‘S’ the only contributor to the experimental variability, though no significant differences emerged in relation to fruit size. Accordingly, for all the taste variables, the scion genotypes proved lower σ^2^_E_ values when compared to rootstocks.

Soluble sugars (mainly glucose, fructose and sucrose) and organic acids (mainly citric and malic) are key taste-compounds of tomato fruits, whose amounts are commonly measured through total soluble solids (TSS) and tritatable acidity (TA), respectively [38,39]. When the organoleptic quality of tomatoes is concerned, their measure is referred to the perceived sweetness (TSS) and sourness (TA), whereas the TSS/TA ratio describes the overall balance among them in the perceived taste [12]. Although fruit itself can partially contribute to carbon fixation, most of this element (85–90%) needed for fruit growth is imported from leaves through the phloem tissue, in the form of carbohydrates [33,40]. In contrast, despite a certain amount of organic acids being able to be supplied through the sap, their accumulation in fleshy fruits is primarily due to the metabolism of citrate and malate in the fruit itself [41,42,43]. In our experiments, both TSS and TA peaked in the small-fruited scions. For TSS this is consistent with its inverse proportionality with the fruit size [39]. However, grafting onto the most vigorous rootstocks (i.e., the *S. habrochaites* and *S. peruvianum* classes) acted to limit TSS, irrespective of the scions’ characteristics. This seems to reinforce the hypothesis of a limited photosynthates flow toward fruits, due to the modified source-sink edaphic relationships induced by vigorous rootstocks [5,27]. Additionally, efficient rootstocks in water absorption may increase fruit water content, leading to a decreased fruit sugars concentration [11,44]. This hypothesis is corroborated by observing that, despite a lower fruit biomass in the most vigorous rootstocks, no grafting effect was recorded on scions’ yield, likely deriving from an increased fruit size due to a higher water content. On the contrary, all the rootstocks under study promoted TA in small-fruited scions, generating a lower TSS/TA ratio, especially in the *S. habrochaites* and *S. peruvianum* hybrid rootstocks (−8%, on average). Organic acids (in particular citrate) tend to substitute sugars as a respiratory substrate in the case of cytosolic carbohydrates shortage into the fruit [45,46]. This hypothesis seems to be corroborated by the highest TA increase in response to grafting recorded in the small-fruited scions, i.e., those most suffering the sink strength imposed by the rootstocks.

Beyond its importance as antioxidant in the human diet [47] ascorbic acid (AsA) content plays a pivotal role for the plant, being involved in cell division, cell wall synthesis and in the interaction of the plant with the surrounding environment [48]. According to Massot et al. [49] the AsA concentration in tomatoes is the complex result from its import (or the import of precursors) from leaves, its synthesis and recycling inside the fruit and its export outside the fruit. In the present study, the AsA content proved to be mostly under the scion influence, but when these were clustered in the ANOVA, it was impossible to establish differences on the basis of the fruit size. Accordingly, there were huge differences among scions in terms of σ^2^_E_, whose highest values were recorded both in large-fruited (‘Porpora’) and small-fruited scions (‘Caprice’ and ‘Beka’). In contrast, despite the significant ‘S × R’ interaction, the ANOVA for rootstock classes revealed that all the heterograft combinations dramatically lowered the fruit AsA content, particularly in the highly vigorous *S. habrochaites* hybrids (−45%). The lowest fruit AsA content characterizing the most vigorous grafting combinations has been explained through their higher vegetative biomass, resulting in a redistribution or accumulation of this metabolite in other plant fractions [50]. However, this hypothesis can only partially explain our data, since the low fruit AsA content recorded within the *S. pimpinellifolium* class (i.e., the least vigorous one). The analysis of correlation (data not shown) revealed that fruit weight and AsA content were negatively related (−0.969 *), indicating the existence of a dilution effect altering this important compositional trait even in the least vigorous rootstocks.

## 5. Conclusions

The outcome of this experiment, conducted on a large set of cherry tomato graft combinations, highlighted variable responses in terms plant growth, yield and fruit quality traits. Among the 16 variables reported, the scion genotype per se influenced cherry tomatoes appearance (L*, b* and shape index) and taste (TSS and the TSS/TA ratio). This implies that the scion cultivar was the top contributor to those traits mostly related to the commercial identity of the fruit. On the other hand, the broader significance of the ‘S × R’ interaction (for 10 out of 16 variables) suggested that, when graft partners were analyzed individually, the bio-agronomical response of cherry tomato to grafting was largely unpredictable, most of all in terms of plant growth and yield performances. However, when both scions and rootstocks were clustered by class, some of these responses were clearly attributable to the intrinsic fruit size of the scion and/or to the genetic background of the rootstock. The *S. habrochaites* rootstocks maximized the plant biomass, most of all in terms of vegetative organs, whereas the *S. pimpinellifolium* ones did the opposite, but both classes significantly reduced the fruit biomass, especially in small- and medium-fruited scions. This would suggest the opportunity to opt for large-fruited cherry tomato cultivars in order to buffer possible yield reductions, at least in non-stressed growth conditions. Where fruit quality was concerned, the *S. habrochaites* and *S. peruvianum* hybrids (i.e., the most vigorous ones) reduced the red hue (a*) and TSS, while promoting TA, thus potentially increasing the overall perceived sourness of the fruits. All these modifications were likely related to a shift in the source:sink edaphic relationships and to an increased water content of the fruits. A significant reduction of the fruit AsA content was always recorded in response to grafting, even in the *S. pimpinellifolium* group, indicating, in the low vigor rootstocks too, the existence of a dilution effect worsening this pivotal nutraceutical trait of tomato fruits.

## Figures and Tables

**Table 1:** Scion and rootstock genotypes used in the experiment, grouped on the basis of their fruit size and genetic background, respectively.

**Scion**	**Fruit Size**	**Seed Company**
‘Porpora’	Large	Esasem
‘Creativo’	Medium	HM Clause
‘5525′	Medium	Axia Vegetable Seeds
‘Dreamer’	Medium	Nunhems
‘Eletta’	Medium	Top Seeds
‘Beka’	Small	Top Seeds
‘Caprice’	Small	Top Seeds
**Rootstock**	**Genetic Background**	**Seed Company**
‘Optifort’	*S. lycopersicum* × *S. habrochaites*	De Ruiter-Bayer
‘Kaiser’	*S. lycopersicum* × *S. habrochaites*	Rijk Zwaan
‘Mozart’	*S. lycopersicum* × *S. habrochaites*	Royal Seeds
‘Interpro’	*S. lycopersicum* × *S. habrochaites*	Vilmorin
‘Bental’	*S. lycopersicum* × *S. peruvianum*	Top Seeds
‘Pittam’	*S. lycopersicum* × *S. peruvianum*	Top Seeds
‘Dynafort’	*S. lycopersicum* × *S. pimpinellifolium*	De Ruiter-Bayer
‘Tawa’	*S. lycopersicum* × *S. lycopersicum*	Top Seeds

**Table 2:** Summary of the main results of the analysis of variance of the main factors and their interaction for the bio-agronomical and qualitative variables, with the corresponding significance of the *F*-values.

Variable	Scion (S)			Rootstock (R)			S × R		
	Variance	SS_%_	*F*-Test	Variance	SS_%_	*F*-Test	Variance	SS_%_	*F*-Test
Plant biomass	7772.096	55.9	***	1944.341	17.2	***	3238.682	19.8	***
Vegetative biomass	5472.631	46.0	***	2645.018	25.5	***	3270.876	22.9	***
Fruit biomass	282.221	23.7	***	113.122	14.0	***	606.002	36.5	***
Harvest index	0.010	17.5	***	0.014	33.7	***	0.002	34.7	***
Fruit yield	7036.4	77.6	***	11.8	5.7	***	3.2	9.2	***
Fruits per plant	90242.7	82.9	***	1366.4	1.7	**	874.4	6.4	**
Mean fruit weight	1125.7	89.4	***	12.9	1.4	***	5.9	3.7	**
L*	21.4	33.4	***	2.3	4.8	NS	1.6	20.3	NS
a*	88.6	47.0	***	8.3	5.9	NS	16.0	1.9	**
b*	47.0	44.7	**	1.9	0.5	NS	22.1	1.0	NS
Fruit firmness	9.4	3.2	***	1.2	0.5	NS	8.0	0.6	NS
Shape index	51.1	28.2	***	2.9	1.5	NS	13.2	1.1	NS
Total soluble solids (TSS)	47.1	32.6	***	14.8	9.9	***	8.1	0.9	NS
Tritatable acidity (TA)	45.2	40.2	***	2.2	1.4	NS	14.7	1.6	*
TSS/TA	55.2	47.5	***	5.3	3.9	***	10.2	1.2	NS
L-ascorbic acid content	60.2	56.2	***	34.8	26.1	***	31.8	4.0	***

SS_%_: percentage incidence on sum of squares; NS = not significant; *, **, *** significant at *p* < 0.05, 0.01 and 0.001, respectively.

**Table 3:** Mean and environmental variance (σ^2^_E_) for the different variables and the scions under study. Different letters among means (within each row) indicate significance at Tukey’s HSD test (*p* ≤ 0.05).

Variable	Large-Fruited		Medium-Fruited								Small-Fruited			
	‘Porpora’		‘5525′		‘Creativo’		‘Dreamer’		‘Eletta’		‘Beka’		‘Caprice’	
	Mean	σ^2^_E_	Mean	σ^2^_E_	Mean	σ^2^_E_	Mean	σ^2^_E_	Mean	σ^2^_E_	Mean	σ^2^_E_	Mean	σ^2^_E_
Plant biomass (g DW plant^−1^)	1109 a	5911.0	827 e	1471.8	957 c	7258.5	902 d	4288.2	1006 b	8515.0	933 c	6550.4	899 d	2286.3
Vegetative biomass (g DW plant^−1^)	773 a	6693.4	542 e	1593.5	629 c	8250.4	599 d	5703.6	707 b	9141.2	639 c	7666.6	600 d	2362.5
Fruit biomass (g DW plant^−1^)	336 a	1083.5	285 c	208.6	328 a	337.5	303 b	903.7	299 bc	387.7	294 bc	185.9	299 bc	1927.1
Harvest index (adimensional)	0.31 bc	0.001	0.34 a	0.000	0.35 a	0.001	0.34 a	0.002	0.30 c	0.001	0.32 b	0.001	0.33 a	0.002
Fruit yield (t ha^−1^)	145.9 a	91.2	123.3 c	50.9	135.2 b	38.1	130.9 b	47.8	124.7 c	44.7	103.6 d	8.511	101.8 d	55.6
Fruits per plant (n.)	228 c	264.8	259 b	401.0	256 b	95.7	272 b	44.1	273 b	209.1	376 a	377.2	368 a	812.4
Mean fruit weight (g)	30.5 a	3.499	20.9 c	3.223	22.5 b	2.938	21.1 bc	1.715	21.6 bc	3.452	12.0 d	0.792	11.9 d	0.468
L* (relative unit)	38.3 d	1.168	40.7 a	0.655	39.1 bc	0.497	39.7 b	0.775	38.4 cd	0.411	38.6 cd	0.332	38.3 d	0.208
a* (relative unit)	12.0 cd	0.646	12.3 c	2.303	11.0 de	2.307	10.3 e	3.127	13.5 b	1.332	14.9 a	1.080	15.0 a	0.673
b* (relative unit)	18.3 ab	1.200	19.2 a	0.148	18.5 ab	0.170	19.1 a	0.513	18.0 b	0.265	18.4 ab	0.439	18.3 ab	0.707
Fruit firmness (N)	835 bc	8238.9	1232 a	3787.1	1127 a	27787.0	855 bc	7802.4	830 bc	5416.3	910 b	15829.3	764 c	1624.4
Shape index (adimensional)	0.87 ab	0.000	0.83 c	0.000	0.86 b	0.000	0.88 a	0.000	0.87 ab	0.000	0.86 c	0.000	0.86 c	0.000
TSS (°Brix)	4.9 cd	0.192	4.8 d	0.063	5.3 b	0.152	5.0 bd	0.193	5.3 bc	0.168	6.0 a	0.164	6.3 a	0.081
TA (mg CA kg^−1^ FW)	4.0 b	0.032	4.5 a	0.092	4.1 b	0.150	3.7 b	0.120	3.1 c	0.059	4.8 a	0.250	4.9 a	0.156
TSS/TA (adimensional)	1.25 c	0.009	1.08 d	0.005	1.31 bc	0.004	1.36 b	0.002	1.73 a	0.014	1.25 c	0.014	1.30 bc	0.020
AsA (mg kg^−1^ FW)	196 bc	6891.5	194 bc	695.7	171 c	1001.5	174 c	813.5	227 a	1313.6	211 ab	1623.3	206 ab	3437.9

**Table 4:** Mean and environmental variance (σ^2^_E_) for the different variables and the rootstocks under study. Different letters among means (within each row) indicate significance at Tukey’s HSD test (*p* = 0.05).

Variable	*S. lycopersicum* × *S. habrochaites*								*S. lycopersicum* × *S. peruvianum*				*S. lycopersicum* × *S. pimpinellifolium*		*S. lycopersicum* × *S. lycopersicum* Ungrafted			
	‘Optifort’		‘Kaiser’		‘Mozart’		‘Interpro’		‘Bental’		‘Pittam’		‘Dynafort’		‘Tawa’		Ungrafted	
	Mean	σ^2^_E_	Mean	σ^2^_E_	Mean	σ^2^_E_	Mean	σ^2^_E_	Mean	σ^2^_E_	Mean	σ^2^_E_	Mean	σ^2^_E_	Mean	σ^2^_E_	Mean	σ^2^_E_
Plant biomass (g DW plant^−1^)	1005 a	11239.4	1007 a	14604.6	955 bc	17081.0	990 ab	11683.6	939 cd	7113.8	955 bc	17695.3	867 f	3675.0	918 de	9126.9	892 ef	6877.3
Vegetative biomass (g DW plant^−1^)	710 a	7058.7	709 a	13472.5	665 bc	13047.8	688 ab	11802.6	629 d	6684.9	641 cd	13764.1	574 e	2984.3	590 e	7016.1	566 e	2860.7
Fruit biomass (g DW plant^−1^)	295 bc	1277.5	298 bc	471.8	291 c	609.9	302 bc	1095.8	311 ac	1240.1	314 ab	586.6	292 c	489.3	328 a	555.8	326 a	1667.1
Harvest index (adimensional)	0.29 c	0.001	0.30 c	0.001	0.31 c	0.001	0.31 c	0.002	0.33 b	0.002	0.33 b	0.001	0.34 b	0.001	0.36 a	0.001	0.36 a	0.001
Fruit yield (t ha^−1^)	128.3 a	491.0	125.5 ab	211.1	122.8 ac	274.3	121.7 bd	301.3	125.8 ab	342.1	126.7 ab	321.1	116.2 d	256.6	127.6 a	310.5	117.9 cd	114.3
Fruits per plant (n.)	288 ab	2644.1	293 ab	5990.3	282 ab	3258.7	289 ab	4675.5	290 ab	3932.3	298 a	3416.1	275 b	2513.9	298 a	1963.6	300 a	4018.0
Mean fruit weight (g)	20.4 a	43.0	20.7 a	57.4	20.4 a	30.5	20.4 a	47.2	20.5 a	46.8	20.3 a	44.2	20.0 a	49.4	19.9 a	35.1	18.1b	37.4
L* (relative unit)	39.1 a	1.029	38.9 a	1.963	39.7 a	1.166	38.7 a	1.889	38.8 a	1.153	39.4 a	1.610	38.7 a	1.085	39.1 a	1.234	38.8 a	0.322
a* (relative unit)	12.2 bc	2.443	12.4 bc	4.067	11.8 c	4.228	13.0 ac	2.785	12.3 bc	8.490	12.1 c	6.530	13.2 ac	3.649	14.1 a	4.514	13.5 ab	2.890
b* (relative unit)	18.5 a	1.271	18.3 a	0.400	18.9 a	0.831	18.3 a	0.473	18.4 a	0.580	18.6 a	0.796	18.7 a	0.617	18.5 a	0.582	18.6 a	0.468
Fruit firmness (N)	934 a	29956.8	903 a	25969.2	942 a	51700.9	896 a	39297.0	894 a	24298.0	959 a	41692.8	915 a	36557.0	952 a	36384.4	1030 a	65323.2
Shape index (adimensional)	0.85 a	0.001	0.85 a	0.001	0.85 a	0.000	0.86 a	0.001	0.86 a	0.001	0.85 a	0.001	0.85 a	0.001	0.85 a	0.001	0.85 a	0.001
TSS (°Brix)	4.98 d	0.503	4.98 d	0.278	5.10 cd	0.385	5.47 ac	0.398	5.33 bd	0.308	5.41 ad	0.380	5.50 ac	0.348	5.81 a	0.315	5.76 ab	0.231
TA (mg CA kg^−1^ FW)	3.89 a	0.608	4.05 a	0.591	4.13 a	0.708	4.33 a	0.450	4.21 a	0.336	4.14 a	0.404	4.21 a	0.523	4.23 a	0.673	4.18 a	0.466
TSS/TA (adimensional)	1.31 ac	0.032	1.27 c	0.031	1.28 c	0.060	1.29 ac	0.036	1.29 bc	0.030	1.33 ac	0.032	1.35 ac	0.053	1.42 ab	0.060	1.43 a	0.080
AsA (mg kg^−1^ FW)	159 c	903.3	167 bc	1191.7	189 b	608.1	178 bc	863.6	187 bc	445.4	196 b	235.2	194 b	885.9	239 a	1664.3	266 a	6812.8

**Table 5:** Plant growth, developmental and yield variables of cherry tomato as affected by scion and rootstock class (main effects). Different letters within each column’s factor indicate significance at Tukey’s HSD test (*p* = 0.05).

Source of Variation	Plant Biomass	Vegetative Biomass	Fruit Biomass	Harvest Index	Fruit Yield	Fruits per Plant	Mean Fruit Weight
	(g DW Plant^−1^)	(g DW Plant^−1^)	(g DW Plant^−1^)	(Adimensional)	(t ha^−1^)	(n Plant^−1^)	(g FW)
Scion class							
Large fruit	1079 a	738 a	341 a	0.32 a	144.3 a	228 c	30.4 a
Medium fruit	896 a	588 a	307 a	0.35 a	127.1 b	268 b	21.0 b
Small fruit	899 a	595 a	303 a	0.34 a	102.6 c	369 a	11.9 c
Rootstock class							
*S. lycopersicum* × *S. habrochaites*	1023 a	722 a	301 b	0.30 b	126.3 a	287 a	21.6 a
*S. lycopersicum* × *S. peruvianum*	986 a	666 a	320 a	0.33 ab	129.1 a	293 a	21.7 a
*S. lycopersicum* × *S. pimpinellifolium*	894 b	602 b	292 b	0.33 ab	117.0 a	269 b	21.8 a
*S. lycopersicum* × *S. lycopersicum*	956 ab	623 ab	333 a	0.35 a	130.0 a	298 a	20.8 ab
Ungrafted	930 b	590 b	340 a	0.36 a	120.8 a	295 a	19.7 b
*F*-test							
Scion class (S_class_)	NS	NS	NS	NS	**	***	***
Rootstock class (R_class_)	***	***	***	***	NS	**	**
S_class_ × R_class_	NS	NS	**	NS	NS	*	NS

NS = not significant; *, **, *** significant at *p* < 0.05, 0.01 and 0.001, respectively.

**Table 6:** Carpometric and fruit quality traits of cherry tomato as affected by scion and rootstock class (main effects). Different letters within each column’s factor indicate significance at Tukey’s HSD test (*p* = 0.05).

Source of Variation	L*	a*	b*	Firmness	Shape Index	TSS	TA	TSS/TA	AsA Content
	(Relative Unit)	(Relative Unit)	(Relative Unit)	(g)	(Adimensional)	(°Brix)	(mg CA kg^−1^ FW)	(Adimensional)	(mg kg^−1^ FW)
Scion class									
Large fruit	38.4 a	12.3 a	18.3 a	859 a	0.87 a	5.14 b	4.02 b	1.29 a	226.9 a
Medium fruit	39.4 a	12.1 a	18.7 a	1022 a	0.86 a	5.25 b	3.92 b	1.39 a	202.0 a
Small fruit	38.5 a	15.3 a	18.4 a	844 a	0.83 a	6.23 a	4.77 a	1.32 a	227.1 a
Rootstock class									
*S. lycopersicum* × *S. habrochaites*	38.8 a	12.5 b	18.3 a	867 a	0.85 a	5.13 b	4.18 a	1.25 b	167.8 d
*S. lycopersicum* × *S. peruvianum*	38.8 a	12.5 b	18.5 a	905 a	0.85 a	5.40 b	4.26 a	1.28 b	191.9 c
*S. lycopersicum* × *S. pimpinellifolium*	38.8 a	13.4 ab	18.9 a	903 a	0.85 a	5.49 ab	4.31 a	1.30 ab	191.2 c
*S. lycopersicum* × *S. lycopersicum*	38.7 a	14.0 a	18.2 a	904 a	0.85 a	5.88 a	4.28 a	1.40 a	236.8 b
Ungrafted	38.6 a	13.6 ab	18.5 a	966 a	0.85 a	5.81 a	4.16 a	1.42 a	305.6 a
*F*-test									
Scion class (S_class_)	NS	NS	NS	NS	NS	*	*	NS	NS
Rootstock class (R_class_)	NS	**	NS	NS	NS	***	NS	***	***
S_class_ × R_class_	NS	NS	NS	NS	NS	NS	***	***	NS

NS = not significant; *, **, *** significant at *p* < 0.05, 0.01 and 0.001, respectively.
